# Rainbow‐Colored Carbon Nanotubes via Rational Surface Engineering for Smart Visualized Sensors

**DOI:** 10.1002/advs.202303593

**Published:** 2023-08-27

**Authors:** Jing Zhang, Xueqing Tang, Jie Wei, Shan Cong, Siqi Zhu, Yaowu Li, Jian Yao, Weibang Lyu, Hehua Jin, Meng Zhao, Zhigang Zhao, Qingwen Li

**Affiliations:** ^1^ School of Nano‐Tech and Nano‐Bionics University of Science and Technology of China Hefei 230026 China; ^2^ Key Laboratory of Multifunctional Nanomaterials and Smart Systems Advanced Materials Division Suzhou Institute of Nano‐Tech and Nano‐Bionics Chinese Academy of Sciences Suzhou 215123 China; ^3^ Jiangsu Key Laboratory of Micro and Nano Heat Fluid Flow Technology and Energy Application Suzhou University of Science and Technology Suzhou 215009 China

**Keywords:** carbon nanotubes, hydrogen leakage, localized response, rainbow‐colored, smart sensors

## Abstract

Surface engineering is effective for developing materials with novel properties, multifunctionality, and smart features that can enable their use in emerging applications. However, surface engineering of carbon nanotubes (CNTs) to add color properties and functionalities has not been well established. Herein, a new surface engineering strategy is developed to achieve rainbow‐colored CNTs with high chroma, high brightness, and strong color travel for visual hydrogen sensing. This approach involved constructing a bilayer structure of W and WO_3_ on CNTs (CNT/W/WO_3_) and a trilayer structure of W, WO_3_, and Pd on CNTs (CNT/W/WO_3_/Pd) with tunable thicknesses. The resulting CNT/W/WO_3_ composite film exhibits a wide range of visible colors, including yellow, orange, magenta, violet, blue, cyan, and green, owing to strong thin‐film interference. This coloring method outperforms other structural coloring methods in both brightness and chroma. The smart CNT/W/WO_3_/Pd films with porous characteristics quickly and precisely detect the hydrogen leakage site. Furthermore, the smart CNT/W/WO_3_/Pd films allow a concentration as low as 0.6% H_2_/air to be detected by the naked eye in 58 s, offering a very practical and safe approach for the detection and localization of leaks in onboard hydrogen tanks.

## Graphical abstract

A surface engineering strategy is developed to create rainbow‐colored carbon nanotubes (CNTs) for visual hydrogen sensing. By utilizing bilayer and trilayer structures of W, WO_3_, and Pd on CNTs, a wide range of colors is achieved. The smart CNT/W/WO_3_/Pd films enables rapid and accurate detection of hydrogen leaks by the naked eye, offering a safe leak detection solution for onboard hydrogen tanks.

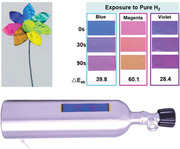

## 1 Introduction

Carbon nanotubes (CNTs) have been widely studied in the past few decades because of their high strength, charge carrier mobility, thermal conductivity, and mechanical flexibility, which can be used for a broad range of applications in mechanics, microelectronics, biomedicine, optoelectronics, and photonics.^[^ ^1^, ^2^, ^3^, ^4^, ^5^, ^6^, ^7^ ^]^ However, like other types of carbon materials, CNTs usually appear black or even super black owing to their extremely strong light absorption, which greatly hinders their use in different applications, such as functional coatings, smart textiles, and decorative composites.^[^ ^8^, ^9^, ^10^, ^11^, ^12^ ^]^ Therefore, there is a strong impetus to develop CNTs with a high color quality while maintaining their excellent intrinsic properties.

The coloration of CNTs using dyes, pigments, and paints has proven to be ineffective because of the insufficient chemical affinity of CNTs for these colorants owing to their highly chemically inert surfaces. Chirality separation of narrow‐distribution CNTs can be used to create multicolored CNTs however, this technique is complicated, time‐consuming, low‐yielding, and generally applicable only to ultrashort single‐walled CNTs.^[^ ^13^, ^14^ ^]^ In contrast, recent research has demonstrated that using the nanostructures on CNTs to create structural colors is an efficient and viable alternative to traditional coloring methods. For instance, Zhang et al. successfully achieved the structural coloration of CNTs using atomic layer deposition of amorphous TiO_2_ layers^[^ ^15^ ^]^ and the self‐assembly of silica photonic crystals.^[^ ^16^ ^]^ This approach and others have expanded the structural and functional possibilities of CNTs. However, previously studied CNTs coloration techniques have notable limitations. The atomic layer deposition method is expensive, time‐consuming, and unsuitable for large‐scale production, and achieving a large‐area programmable assembly of photonic crystals with good structural stability remains a challenging task. Moreover, the color qualities obtained through dyeing methods are often unsatisfactory, particularly in terms of chroma (color purity measured by the CIE gamut) and brightness (reflectance). Therefore, the coloration of CNTs with high quality and efficiency remains a significant challenge.

Furthermore, the colors of CNTs produced by previously studied coloring methods are static and are unable to produce dynamic changes. CNTs with dynamic colors may provide a unique opportunity to design novel structure‐function integrated materials, such as multicolor hydrogen‐sensing materials while maintaining their excellent mechanical properties. As is well known, gasochromic materials or sensors can make invisible hydrogen gas visible to the naked eye without electricity or complex equipment.^[^ ^17^, ^18^, ^19^, ^20^, ^21^, ^22^, ^23^, ^24^ ^]^ Thus, endowing CNTs with stimuli‐responsive chromatic properties may allow these materials to be used in promising applications, such as on CNT‐reinforced hydrogen tanks to provide a visual warning of hydrogen leaks and prevent fires and explosions.

In this study, we present a new coloring approach by constructing a bilayer structure of W and WO_3_ on CNTs (CNT/W/WO_3_) and a trilayer structure of W, WO_3_, and Pd on CNTs (CNT/W/WO_3_/Pd) to form an optical resonant cavity (Fabry–Pérot (F–P) nanocavity), giving the CNTs a wide range of vivid rainbow‐like colors that can be adjusted by adjusting the size of the resonant cavity, allowing the colors to range from yellow to orange, magenta, violet, blue, cyan, and green. This method significantly improves the color intensity of the CNTs, with a maximum increase in chroma and brightness of 53% and 28%, respectively, compared with the existing TiO_2_ coating method. Interestingly, a clear multicolor change that accurately locates hydrogen leaks can be visually identified within 6 s by the naked eye, and concentrations as low as 0.6% H_2_/air can be detected by the naked eye in 58 s. The most significant difference between our color‐changing CNT/W/WO_3_/Pd film and traditional gasochromic films is that the color change is achieved through the direct penetration of the porous structure of the CNT film by the hydrogen gas.^[^ ^25^ ^]^ This material has a fast detection speed, and its multicolor capabilities can be used for different applications.

## 2 Results and Discussion

### 2.1 Surface Engineering Design of Rainbow‐Colored CNTs

**Figure 1:**
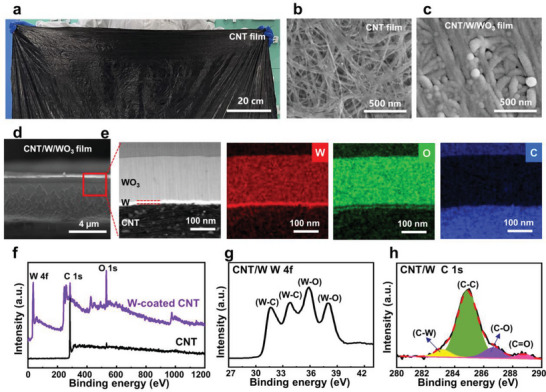
Structural characterizations of colorful CNT films. a) Optical photograph of the pristine CNT film. b) SEM images showing the surface structure of the pristine CNT film. c) The top‐view SEM images of the CNT/W/WO_3_ film. d) The cross‐sectional SEM image of CNT/W/WO_3_. e) TEM images of an enlarged view of the cross‐section structure and the corresponding element mappings of CNT/W/WO_3_. f) XPS spectrum of the pristine CNT film and a W‐coated CNT film (CNT/W). g) XPS spectrum of W 4f for CNT/W. h) XPS spectrum of C 1s for CNT/W.

First, a raw pristine CNT film was synthesized using floating‐catalyst chemical vapor deposition, where absolute ethyl alcohol, ferrocene, and thiophene served as the carbon source, catalyst precursor, and promoter, respectively. The resultant film was 5–10 µm thick and had a length and width of ≈ 1.5 and 0.5 m, respectively. The film was cut into small ribbons for the coloration study. The pristine CNT film appeared dark black, as shown in **Figure** **1**a. Microscopic characterization revealed that the CNT film was composed of a network of densely packed CNTs with diameters of ≈ 5–10 nm (Figure 1b). The top‐view scanning electron microscopy (SEM) image of the CNT/W/WO_3_ film showed that the W/WO_3_ layer was deposited along the network structure of the CNTs and maintained a relatively smooth and homogeneous top surface (Figure 1c). The cross‐sectional SEM image of the CNT/W/WO_3_ film showed a perfect bilayer assemblage consisting of a thin metal W layer (12 nm) and a thin WO_3_ layer (200 nm) on the CNTs, with very small thickness variations (Figure 1d,e). An energy‐dispersive X‐ray spectroscopy (EDX) analysis of the CNT/W/WO_3_ also indicated that a uniform distribution was achieved for the W and WO_3_ layers (Figure 1e). The W‐coated CNT film (CNT/W) was further characterized by X‐ray photoelectron spectroscopy (XPS) (Figure 1f,h). It can be seen that the XPS W4f core level spectrum can be deconvoluted into two pairs of peaks with binding energies of 31.7, 33.7, 35.8, and 37.9 eV. The major peaks with lower binding energies of 31.7 and 33.7 eV can be attributed to the W─C bonds, and the peaks with higher binding energies (35.8 eV for W 4f 7/2 and 37.9 eV for W 4f 5/2) can be attributed to the W─O bonds.^[^ ^26^ ^]^ Similarly, the formation of the W─C bonds was also evidenced by the C 1s XPS spectrum, which showed a broad carbide C 1s signal located at ≈ 283.1 eV. Thus, it can be concluded that there was a strong interaction between the W and CNTs, which may indicate strong adhesion of structural colors to the CNTs. X‐ray diffraction (XRD) analysis of the CNT/W/WO_3_ film showed the presence of tungsten carbide crystalline forms (Figure S1, Supporting Information). Transmission electron microscopy (TEM) was performed to investigate the interfacial properties of the CNT/W/WO_3_ films. The TEM images showed no gaps at the C─W or W─WO_3_ interfaces at the nanometer scale (Figure S2, Supporting Information), indicating good interfacial wettability and tight binding between the different layers. Overall, the XPS and TEM results provide strong evidence of the high quality and integrity of the CNT/W/WO_3_ films.

### 2.2 Rainbow‐Colored CNTs

**Figure 2:**
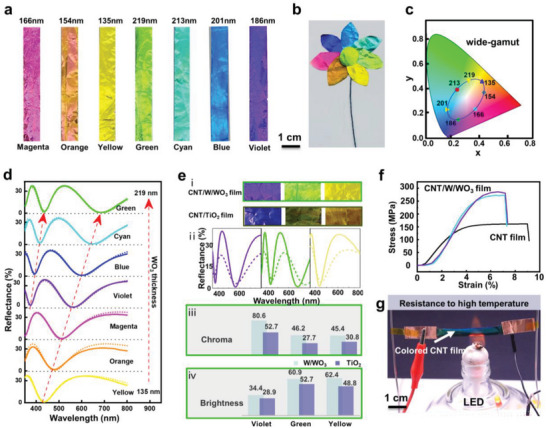
High quality and wide‐gamut coloring of CNT/W/WO_3_ film and its performance. a) Optical images of the CNT/W/WO_3_ films in seven colors. b) Seven colors of CNT/W/WO_3_ films cut into a 7‐color flower. c) The CIE color coordinates for seven colors of CNT/W/WO_3_ films. d) Simulated (dashed lines) and measured (solid lines) reflection spectra with different thickness of WO_3_: 135, 154, 166, 186, 201, 213, and 219 nm. e) Comparison of three groups of similarly colored CNT films dyed with W/WO_3_ and TiO_2_, respectively i) Optical images, ii) Reflectance spectra, CNT/W/WO_3_ (solid lines) and CNT/TiO_2_ (dashed lines), iii) Chroma value, iv) Brightness value. f) Stress–strain curves of CNT/TiO_2_ and CNT/W/WO_3_ films. g) Demonstration of high‐temperature resistance and electrical conductivity of CNT/W/WO_3_ film by lighting an LED when heated in an alcohol lamp.

The resultant CNT/W/WO_3_ films displayed wide‐gamut, high‐brightness, and high‐chroma colors. For instance, by controlling the thickness of the WO_3_ layer to 166, 154, 135, 219, 213, 201, and 186 nm (with the thickness of the underlying metallic W layer fixed at 12 nm), the CNT films can exhibit seven highly saturated colors: magenta, orange, yellow, green, cyan, blue, and violet (**Figure** **2**a). A 7‐petal colorful CNT flower was created using seven different CNT/W/WO_3_ films by simple cutting (Figure 2b). The color gamut of this material can be described by its location within the CIELAB color space (CIE 1931). It can be observed that the color points on the color diagram map form a complete circle, demonstrating the wide color gamut of our colorful CNTs (Figure 2c). Corresponding to the increase in the thickness of the WO_3_ layer, the reflection valley in the reflection spectra shifted accordingly from 435 to 681 nm (Figure 2d). In this design, CNTs, W, and WO_3_ formed an F–P resonant cavity with the optical propagation path, as shown in Figure S3 (Supporting Information). The reflection rate calculation formula is shown in Equations S1–S4 (Supporting Information). The refractive indices of the CNTs, W, and WO_3_ were obtained using a spectroscopic ellipsometer (Figure S4, Supporting Information). The dashed line in Figure 2d represents the calculated reflectance spectra, derived using finite difference time domain (FDTD) calculations.^[^ ^27^ ^]^ The overall shape of each experimental spectrum matched the calculated spectra well for the same WO_3_ thickness when the thickness of the metallic W layer was set to 12 nm. A similar trend was observed in the wavelength reflectance when the thickness of WO_3_ varied. Therefore, by adjusting the thickness of WO_3_, the color of the CNTs can be precisely controlled across the entire color spectrum. Our colorful CNT/W/WO_3_ films shone brighter than those coated with TiO_2_ (Figure 2ei). As shown in Figure 2eii, a higher reflectivity of ≈ 38% was achieved for our colorful CNT/W/WO_3_ films, while the maximum reflectivity obtained for the TiO_2_‐coated CNTs (CNT/TiO_2_) was only ≈ 28% in the visible spectrum. Thus, our colorful CNT/W/WO_3_ films offer chroma values, calculated from the corresponding reflection spectra with a D65 standard light source, that was 47%–67% higher than those of previously reported CNT/TiO_2_ films (Figure 2eiii), indicating a considerable high level of chroma for varied hues in our samples. Furthermore, the color brightness coordinates of our yellow, green, and violet CNT/W/WO_3_ films were 16%–28% higher than those of CNT/TiO_2_ films (Figure 2eiv). Additionally, we compared the effects of the W/WO_3_ coating on the mechanical and electrical properties of the CNT/W/WO_3_ films. Figure 2f shows that the W/WO_3_ coating significantly improves the mechanical strength of the CNT/W/WO_3_ films from 150 to 270 MPa, probably because of the enhanced interaction between the CNT bundles by the metallic W layer. This is crucial for developing structure‐function integrated materials. Figure S5 (Supporting Information) shows that the electrical conductivity of the CNT/W/WO_3_ films was maintained, but decreased slightly as the thickness of the WO_3_ layer increased.

Interestingly, the colorful CNT/W/WO_3_ films exhibited brilliant colors and good conductivity, even under extreme circumstances. After exposure to UV radiation for four months, the colorful CNT/W/WO_3_ film retained the same color sensation, as evidenced by the unchanged reflection spectra (Figure S6, Supporting Information), and the CNT/W/WO_3_ films maintained their vivid structural colors even when subjected to low‐temperature cooling in liquid nitrogen at −196°C (Figure S7, Supporting Information) and high‐temperature heating in an alcohol lamp at a maximum temperature of 600°C (Figure 2g). To characterize the conductivity of the CNT/W/WO_3_ films at high temperatures, we connected a CNT/W/WO_3_ film in series with an LED and applied a certain voltage to illuminate the LED. When we heated the CNT/W/WO_3_ film with an alcohol lamp, we observed that the color of the CNT/W/WO_3_ film and the brightness of the LED did not change. This indicated that the conductivity of the CNT/W/WO_3_ film remained stable at high temperatures (Video S1, Supporting Information). The characterization above confirms that the colored CNT/W/WO_3_ films not only maintain their color but also possess conductivity, mechanical strength, high‐ and low‐temperature resistance, and radiation resistance.

### 2.3 Performance of the Smart Visualized Sensor

**Figure 3:**
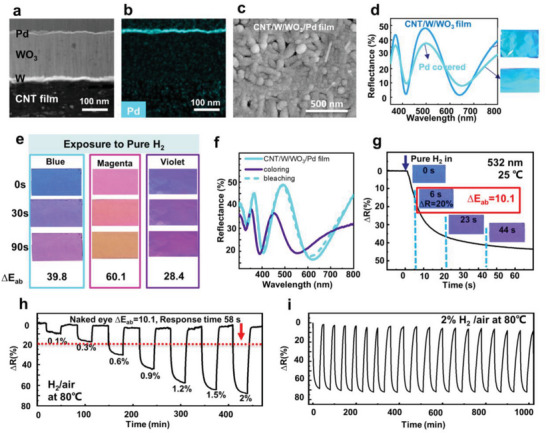
Gas‐chromic properties of CNT/W/WO_3_/Pd. a) TEM image of a cross‐section of CNT/W/WO_3_ covered with a layer of Pd. b) Corresponding element mappings of Pd. c) SEM images of the surface of CNT/W/WO_3_/Pd. d) Reflection spectra and optical images of CNT/W/WO_3_ before and after Pd addition. e) Optical images of color change with time for three different colors of CNT/W/WO_3_/Pd exposed to pure H_2_, and the maximum color difference (ΔE_ab_) before and after color change. f) Reflectance spectra of blue film coloring and bleaching in pure H_2_. g) Changes in reflectance (532 nm) and color with time for blue CNT/W/WO_3_/Pd film exposed to pure H_2_ at 25°C. h) The variation of reflectance change of blue CNT/W/WO_3_/Pd film with coloring‐bleaching cycles at different concentrations of hydrogen gas (H_2_ + Air) at a 532‐nm wavelength and 80°C. i) Coloring‐bleaching cycle of blue CNT/W/WO_3_/Pd film under hydrogen gas (2% H_2_ + 98% Air) and air atmosphere at a 532‐nm wavelength and 80°C.

WO_3_ is considered a promising optical hydrogen sensor for various applications owing to its high optical modulation rate, high coloring efficiency, excellent cycling stability, and low raw material cost.^[^ ^17^, ^18^, ^19^, ^20^, ^21^, ^22^, ^23^, ^24^ ^]^ To achieve smart color tunability for the CNTs, a trilayer structure of W, WO_3_, and Pd layers was deposited in sequence onto the CNTs, creating the CNT/W/WO_3_/Pd structure. This structure included an ultrathin Pd layer with a thickness of ≈ 5 nm sputtered on top of the WO_3_ layer, which acted as a catalyst for hydrogen spillover and storage (**Figure** **3**a).^[^ ^28^ ^]^ The EDX analysis showed that the Pd layer was uniformly distributed (Figure 3b). This addition did not significantly alter the initial surface (Figure 3c), color, or spectral characteristics of the original samples, although a slight decrease in brightness was observed (Figure 3d). In general, the evaluation of the color change in gasochromic films is based on changes in transmittance.^[^ ^18^, ^21^ ^]^ However, in this study, the CNTs completely absorbed visible light, making the entire CNT/W/WO_3_/Pd film opaque. Therefore, we measured the reflectance of the films in all experiments. As reflectance only reflects the parameter related to brightness in the color change, we introduced the color difference (∆E) to better quantify the color change. The value of ∆E can be calculated from the equation: $\Delta \;E_{ab}=\sqrt{{\left(L_{2}^{*}-L_{1}^{*}\right)}^{2}+{\left(a_{2}^{*}-a_{1}^{*}\right)}^{2}+{\left(b_{2}^{*}-b_{1}^{*}\right)}^{2}}$, where L* is the brightness value, a* is the position in the red–green axis, b* is the position in the yellow–blue axis, 1 is the initial state before the color change, and 2 is the state after the color change.^[^ ^29^ ^]^ According to the literature, when ΔE_ab_> 5, the naked eye can see the obvious difference between the two colors.^[^ ^30^, ^31^ ^]^ A possible mechanism for the gasochromic effect of WO_3_ can be explained by charge‐electron transfer between different states. The injection of hydrogen and electrons into the WO_3_ lattice changes the composition of the colored state to a tungsten bronze (H_x_WO_3_), where the degree of coloration of H_x_WO_3_ is closely related to the amount of hydrogen injected (x).^[^ ^28^ ^]^ The literature reports that the value of x for films ranges from 0 to 0.38.^[^ ^32^ ^]^ We simulated and calculated the reflectance curves for three differently colored films using the FDTD method at x values of 0 and 0.38, and found that the degree of blue shift in the reflectance peak was between 30 and 40 nm (Figure S8, Supporting Information). Upon exposure to hydrogen gas, the CNT/W/WO_3_/Pd structure demonstrated a variety of multicolor changes (Figure 3e), and in this experiment, the blue CNT film turned slate blue after 30 s and purple after 90 s, with a maximum ΔE_ab_ value of 39.8. The original magenta film became orange after 90 s of hydrogen gas exposure, with a ΔE_ab_ value of 60.1, while the original violet film turned dark magenta after 90 s of hydrogen gas exposure, with a ΔE_ab_ value of 28.4. Details of the ΔE_ab_ calculations are listed in Table S1 (Supporting Information). These results exceeded the maximum ΔE_ab_ value of 18.5 reported for WO_3_–Pd sensors.^[^ ^19^ ^]^ From the reflection spectra, it can be seen that the presence of hydrogen gas caused a blue shift in the peak position of ≈ 35 nm, and the reflectance also decreased to some extent. This result is consistent with the FDTD method for modeling the effect of hydrogen‐ion insertion. When the film was exposed to air, both the peak level and intensity returned to their original values (bleaching) (Figure 3f; Figure S9, Supporting Information). We tested the performance of commercial WO_3_/Pd gasochromic tapes and observed that pure H_2_ pass‐through changed the tape from yellow to dark blue (Figure S10, Supporting Information). This type of material exhibits only monotonous color modulation from transparent or yellow to blue.^[^ ^17^ ^]^ In contrast, the special structural design of the CNT/W/WO_3_/Pd films can produce rich color tones, which is helpful in meeting the needs of different applications.

To determine the response time of the color change and the detection limit of a hydrogen gas leakage that can be observed with the naked eye, we tested the time‐dependent reflectivity of a blue CNT/W/WO_3_/Pd film at a wavelength of 532 nm in pure H_2_ (Figure 3g). The change in reflectivity was calculated as ΔR (%) = (R_air_−R_H2_)/R_air_ ×100.^[^ ^17^ ^]^ The reflectivity of the film decreased rapidly when the hydrogen gas was introduced; the ΔR value reached 20% after 6 s and then increased to 38 and 42% after 23 and 44 s, respectively. Corresponding to the video of the color change of the film when pure H_2_ was introduced (Video S2, Supporting Information), we calculated the color change of the film by applying hydrogen gas for 6 s, which yielded a ΔE_ab_ value of 10.1, which is much larger than 5, the value required to observe a color change with the naked eye (details of the E_ab_ calculations are given in Table S2, Supporting Information). To further investigate the sensitivity of the film to different hydrogen gas concentrations, we tested the coloring and bleaching abilities of the blue CNT/W/WO_3_/Pd film with seven different hydrogen gas concentrations of 0.1, 0.3, 0.6, 0.9, 1.2, 1.5, and 2% using air as the carrier gas (H_2_/air) at 80 °C (the test setup is shown in Figure S11, Supporting Information; more details see^[^ ^33^ ^]^). As shown in Figure 3h, the value of the reflectivity change at a 532‐nm wavelength gradually increased as the hydrogen gas concentration increased, and the maximum ΔR value was 70%. When the ΔR value was > 20%, the ΔE_ab_ value was > 10.1, indicating that 0.6% H_2_/air could be clearly observed by the naked eye within 58 s. The calculated response times for coloring and bleaching in terms of the time required to achieve a 90% change (ΔR_90%_) were 438 and 156 s, respectively, in 0.6% H_2_/air. The response times for the other concentrations are listed in Table S3 (Supporting Information). Only 6 s were required for the naked eye to recognize the color change in 2% H_2_/air. Therefore, compared with the N_2_ background experiment, the response time of the sensor working in the air was higher, and the response signal was smaller.^[^ ^17^ ^]^ The reversibility and stability of the coloration process were assessed by measuring the reflection spectra of the blue CNT/W/WO_3_/Pd film as a function of time over 18 coloring‐bleaching cycles (Figure 3i). After 18 coloring‐bleaching cycles in 2% H_2_/air, the response amplitude remained at ≈ 70%, whereas the coloring‐bleaching time increased only slightly. This indicates that the colored CNT/W/WO_3_/Pd films exhibited good gasochromic reversibility and stability.

### 2.4 Demonstration of Local Detection of Hydrogen Leaks by the Colorful CNTs

**Figure 4:**
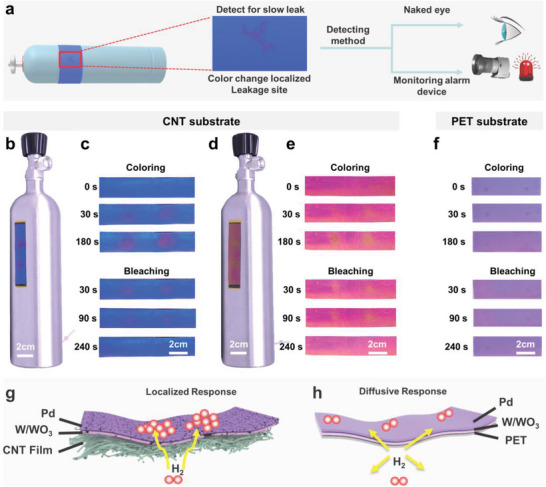
The color changes of colorful films in response to leaking hydrogen tank. a) Schematic diagram of hydrogen gas from leak to detection method. Optical photographs are taken at 180 s after the hydrogen leak, showing b) the blue CNT/W/WO_3_/Pd film and d) the magenta CNT/W/WO_3_/Pd film. Optical photographs of color change during hydrogen leak (coloring) and recovery (bleaching) are presented for c) the blue CNT/W/WO_3_/Pd film, e) magenta CNT/W/WO_3_/Pd film, and f) PET/W/WO_3_/Pd film. Furthermore, the diffusion path on a colorful film during hydrogen leak is illustrated with a schematic diagram for g) the CNT/W/WO_3_/Pd film (localized response) and h) PET/W/WO_3_/Pd film (diffusive response).

Color‐tunable gasochromic films are useful when contrast differentiation is required. As shown in **Figure** **4**a, the CNT/W/WO_3_/Pd films, with excellent mechanical strength and flexibility, could be wrapped around a hydrogen tank or cylindrical connections that are prone to slow leakage. If a microcrack exists in the hydrogen tank, the smart‐colored film can quickly change color at the leakage point. Leaks can be observed with the naked eye or by installing monitoring equipment and alarms. In this study, we conducted an experiment with gasochromic films using a slowly leaking hydrogen tank. Two 3‐mm holes were drilled into the hydrogen tank to simulate a gas leak. Figure 4b–e shows the gasochromic properties of the CNT/W/WO_3_/Pd film. As shown in Figure 4b, when a leak occurs, only the positions of the holes change color from blue to purple, while the surrounding areas remain unchanged, which helps the observers or detection equipment quickly locate the slow leak. Figure 4c shows a magnified view of the film, which shows that a leak of 30 s produces an obvious color change with a ΔE_ab_ value of 29.1. After 180 s of leakage, the color completely changed with a ΔE_ab_ value of 43. When the CNT/W/WO_3_/Pd film was replaced with a PET/W/WO_3_/Pd film under the same conditions (as shown in Figure 4f), the film did not show an obvious color change color after 30 s of leakage, the ΔE_ab_ value was only 10.1 after 180 s, and the position of the leak could not be located (see Table S4, Supporting Information, for data details). Figure 4d,e shows the color change of the magenta CNT/W/WO_3_/Pd film, from magenta to orange, with a ΔE_ab_ value of 44.5 after 180 s of leakage, which also clearly locates the leakage position. The bleaching of the blue and magenta CNT/W/WO_3_/Pd films was rapid. After the hydrogen leakage stopped for 30 s, the color‐change area indicating the leak location decreased significantly, and after 240 s, the film completely returned to its original color. By contrast, the PET/W/WO_3_/Pd film faded to a lesser extent.

The colorful CNT/W/WO_3_/Pd film has significant advantages over conventional hydrogen sensors in terms of response speed and accurate leak localization. This is due to the remarkable flexibility of CNT films, which can closely fit any substrate shape. Additionally, the multilevel pore structure of the underlying CNT films, in which over 90% of its volume is made up of voids, provides an easy pathway for hydrogen to escape from the leakage site to the upper chromic layers. The fluent transportation of hydrogen across the CNT films enables a fast and accurate position identification of the hydrogen leak using a dynamic color change at the leak locations, as depicted in Figure 4g. In contrast, for the dense substrates of PET films, direct transport is hindered and hydrogen molecules must diffuse from the leak location to the random surroundings, leading to ineffective detection or inaccurate localization of hydrogen leaks, as illustrated in Figure 4h. Therefore, the colorful CNT/W/WO_3_/Pd film offers a simple and time‐saving solution for the prompt detection of hydrogen leaks compared to point measurements obtained from electrochemical hydrogen sensors.

## 3 Conclusion

In summary, we proposed a new approach for constructing flexible and tunable full‐color variable films using a two‐layer or three‐layer structure of W, WO_3_, and Pd to create F–P resonant cavities with CNTs. The CNT/W/WO_3_ and CNT/W/WO_3_/Pd films have a high brightness and color saturation and can cover the full color gamut. The CNT/W/WO_3_/Pd colorful films exhibited rich and significant color changes for slowly leaking hydrogen gas. Owing to their excellent multi‐level pore structure, they can achieve fast and accurate naked‐eye localization of hydrogen gas leaks, filling the gap of traditional hydrogen gas sensors, which are insensitive to slow hydrogen leaks. These smart‐colored films also possess excellent mechanical and electrical properties and extreme environmental tolerance. In conclusion, this study provides a reliable method for the development of CNT‐based materials with interesting properties and potential applications.

## 4 Experimental Section

### Fabrication of Colorful CNT Films

The CNT film was synthesized using the floating‐catalyst chemical vapor deposition method with a thickness of ≈ 5–10 µm. First, CNTs were prepared in the form of aerogels, which are ≈ 5 cm thick, free‐standing, but have weak mechanical properties, then the CNT aerogel was placed on a flat plate, sprayed with alcohol to wet the surface, and then the CNT aerogel was rolled into a film with a thickness of 5–10 µm using a rolling method. The mechanical properties were retained after rolling into CNT film. The film was washed with deionized water and then dried at 100°C for 2 h before being cut into 1×10 cm ribbons using a knife. 1) For the CNT/W/WO_3_ films, tungsten and tungsten oxide were deposited layer by layer onto the CNT film using magnetron sputtering (MSP‐620, Jinsheng Instruments, Inc., Beijing). The process involved depositing a 12‐nm metal W layer by sputtering a W target (99.99%) at 200 W and a pressure of 0.3 Pa in an argon atmosphere. Then, the WO_3_ films were deposited by sputtering a W target (99.99%) at 100 W and a pressure of 2.0 Pa in a mixed atmosphere of argon and oxygen. Additional preparation details are presented in Table S5 (Supporting Information). The thickness of the film was determined using cross‐sectional TEM. 2) For the CNT/TiO_2_ films, TiO_2_ was deposited onto the CNT films using an optical thin‐film coater (OTFC‐900, Optorun Co., Ltd.). The TiO_2_ matrix was vapor‐deposited onto the CNT film at a baking temperature of 200°C, which was maintained for both the deposition preparation and process and an evaporation rate of 2 Å s^−1^. Further preparation details are presented in Table S6 (Supporting Information). 3) For the smart color‐tunable films, a Pd layer was deposited on top of the CNT/W/WO_3_ layers by magnetron sputtering (Kurt J. Lesker, Lab 18). A 5‐nm metal Pd layer was sputtered from a Pd target (99.99%) at 300 W and a pressure of 8 × 10^−6^ Pa.

### Characterizations

Optical photos and videos were captured using a Canon EOS 80D camera. SEM images were obtained using an S‐4800 FE‐SEM (Hitachi, Japan) at an acceleration voltage of 5 kV, and cross‐sectional images of the samples were obtained by cutting with a precision cross‐sectional system (Ilion+, Model 693, Gatan) under the protection of liquid nitrogen. To expose the cross‐sectional surface, the colorful films were prepared using a focused ion beam (FIB) microscope (FEI; Thermo Scientific Helios 5UX, USA). Morphological and EDS analyses of the samples were performed using a Tecnai G2 F20 S‐Twin TEM. XPS was performed by an ESCALAB 250Xi XPS instrument with Al‐Kα X‐ray as the excitation source. XRD patterns were recorded on a Bruker D8 Advance powder X‐ray diffractometer at a scanning rate of 4° min^−1^ using Cu–Kα radiation (λ = 1.54056 Å).

### Measurements

The reflectance spectra of the films were measured using a UV–vis spectrophotometer (V660, JASCO) equipped with an integrating sphere measurement system. A spectroscopic ellipsometer (RC2 XI, J. A. Woollam Co., Inc.) was used to measure the optical constants of all materials. The tensile stress–strain curves of the CNT films were obtained using a tensile testing machine Instron 3365 with a gauge length of 10 mm at a loading rate of 1 mm min^−1^. The current–voltage curves of the CNT films were obtained using a CHI660E electrochemical workstation (CH Instruments, Inc.). To assess the conductivity stability of the colored films at high temperatures, the colored carbon film and LED were connected in series with a power supply, and the brightness of the LED was observed over time while the alcohol lamp was lit, and a voltage of 2.5 V was applied. The UV radiation resistance test was conducted by irradiating the films with a 325‐nm UV laser for 4 months at a power of 625 W m^−2^. The color difference was computed using a Python‐based color space conversion library to transform the reflectance spectra of the colors into their corresponding chroma coordinates in the XYZ color space. Subsequently, the L*a*b* value of the sample under the D65 illuminant was determined, and the appropriate formula was applied to evaluate the color difference between the two samples.

### Hydrogen Sensing Test

Hydrogen sensing tests were conducted using a custom‐made optical test system, as shown in Figure S11 of the reference. A 532‐nm laser was used to measure the reflectivity changes during the cycling test at different concentrations and coloring‐bleaching responses. The test gas was adjusted to contain a concentration of 0.1%–2% H_2_/air mixture, and the balance gas was air. For the pure H_2_ discoloration and hydrogen leakage experiments, a hydrogen generator was used to provide hydrogen and natural air was used as the balance gas. The time was recorded using a stopwatch. The coloring start time was measured from the time the hydrogen generator switch was turned on until the first bubble appeared, whereas the fading start time was measured from the time the hydrogen generator switch was turned off until no additional bubbles were observed. The hydrogen tank leakage demonstration experiment consisted of drilling a 3‐mm hole at the bottom of the hydrogen cylinder to allow hydrogen to flow in. Two additional 3‐mm holes were drilled on the opposite side of the bottom hole to act as leak points.

## Conflict of Interest

The authors declare no conflict of interest.

## Supporting information

Supporting Information

Supplemental Video 1

Supplemental Video 2

## Data Availability

Research data are not shared.
