# Supplementary material for: Rainbow‐Colored Carbon Nanotubes via Rational Surface Engineering for Smart Visualized Sensors
Source: Adv Sci (Weinh). 2023 Aug 27;10(29):2303593. doi: 10.1002/advs.202303593 (PMC10582442; doi:10.1002/advs.202303593)
Supplement: Supplementary file 1 — Supporting Information [file ADVS-10-2303593-s001.pdf]

ADVANCED SCIENCE Open Access

Supporting Information

for *Adv. Sci.*, DOI 10.1002/advs.202303593

Rainbow-Colored Carbon Nanotubes via Rational Surface Engineering for Smart Visualized Sensors

Jing Zhang, Xueqing Tang, Jie Wei, Shan Cong, Siqi Zhu, Yaowu Li, Jian Yao, Weibang Lyu, Hehua Jin, Meng Zhao, Zhigang Zhao and Qingwen Li**

Supporting Information

Rainbow-colored carbon nanotubes via rational surface engineering for smart visualized sensors

Jing Zhang#, Xueqing Tang#, Jie Wei, Shan Cong, Siqi Zhu, Yaowu Li, Jian Yao, Weibang Lyu, Hehua Jin, Meng Zhao, Zhigang Zhao, Qingwen Li**

Supplementary Figures

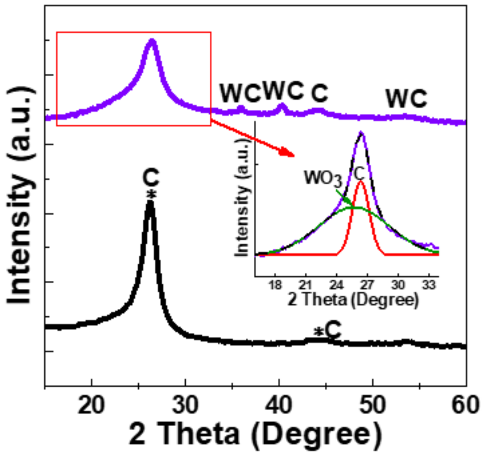

Figure S1. XRD traces of $CNT/W/WO_3$ and CNT films.

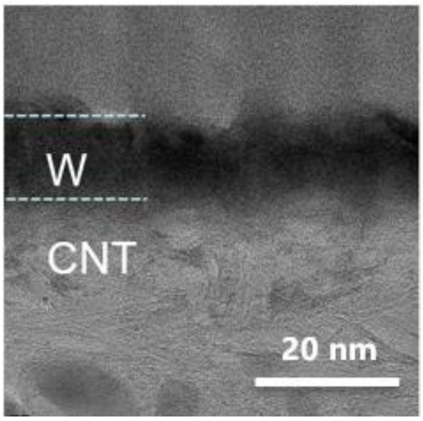

Figure S2. Enlarged TEM images of the cross-sectional structure of $CNT/W/WO_3$ film.

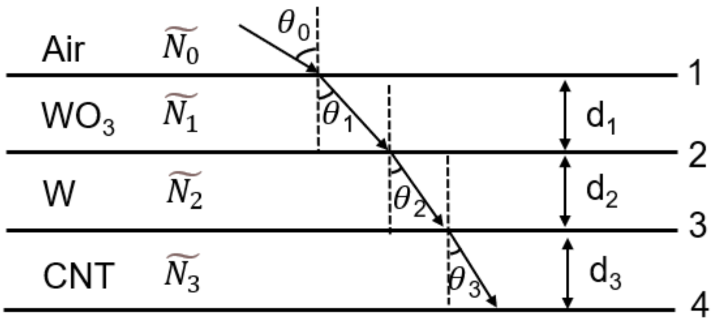

Figure S3. Schematic of light propagation in the CNT/W/WO_3 film. For a simple case of normally incident light, the reflectance in a CNT/W/WO_3 film can be described by equations (S1–S4).

$$\mathrm{R} = \tilde{r}\tilde{r}^* = \left(\frac{\tilde{\varepsilon}_0 B - C}{\tilde{\varepsilon}_0 B + C}\right)\left(\frac{\tilde{\varepsilon}_0 B - C}{\tilde{\varepsilon}_0 B + C}\right)^* \qquad \text{(equation S1)}$$

$$\begin{bmatrix} B \\ C \end{bmatrix} = \left\{\prod_{i=1}^{3}\begin{bmatrix} cos\delta_i & \frac{j}{\tilde{\varepsilon}_i} sin\delta_i \\ j\tilde{\varepsilon}_i sin\delta_i & cos\delta_i \end{bmatrix}\right\}\begin{bmatrix} 1 \\ \tilde{\varepsilon}_3 \end{bmatrix} \qquad \text{(equation S2)}$$

$$\tilde{\varepsilon}_i = \begin{cases} \frac{\widetilde{N}_i}{cos\theta_i} & (p\ polarization) \\ \widetilde{N}_i cos\theta_i & (s\ polarization) \end{cases} \qquad \text{(equation S3)}$$

$$\delta_i = \frac{2\pi}{\lambda}\widetilde{N_\iota} d_i cos\theta_i \qquad \text{(equation S4)}$$

$\widetilde{N_\iota}$ (i = 0,1,2,3) is the complex refractive index of air, the WO_3 layer, the W layer and the CNT film, respectively, θ_i (i= 0,1,2,3) is the incident angle of each layer, d_i (i=1,2,3) is the thickness of the WO_3 and metal W, and λ is the wavelength.

where $\delta_i = \frac{2\pi}{\lambda}\widetilde{N_\iota} d_i cos\theta_i$ and $\tilde{\varepsilon}_i = \begin{cases} \frac{\widetilde{N}_i}{cos\theta_i} & (p\ polarization) \\ \widetilde{N}_i cos\theta_i & (s\ polarization) \end{cases}$ are optical path difference and effective admittance, respectively, and $N_i = n_i - jk_i$ $\widetilde{N_\iota}$ is the complex refractive index. As illustrated by the above equations.

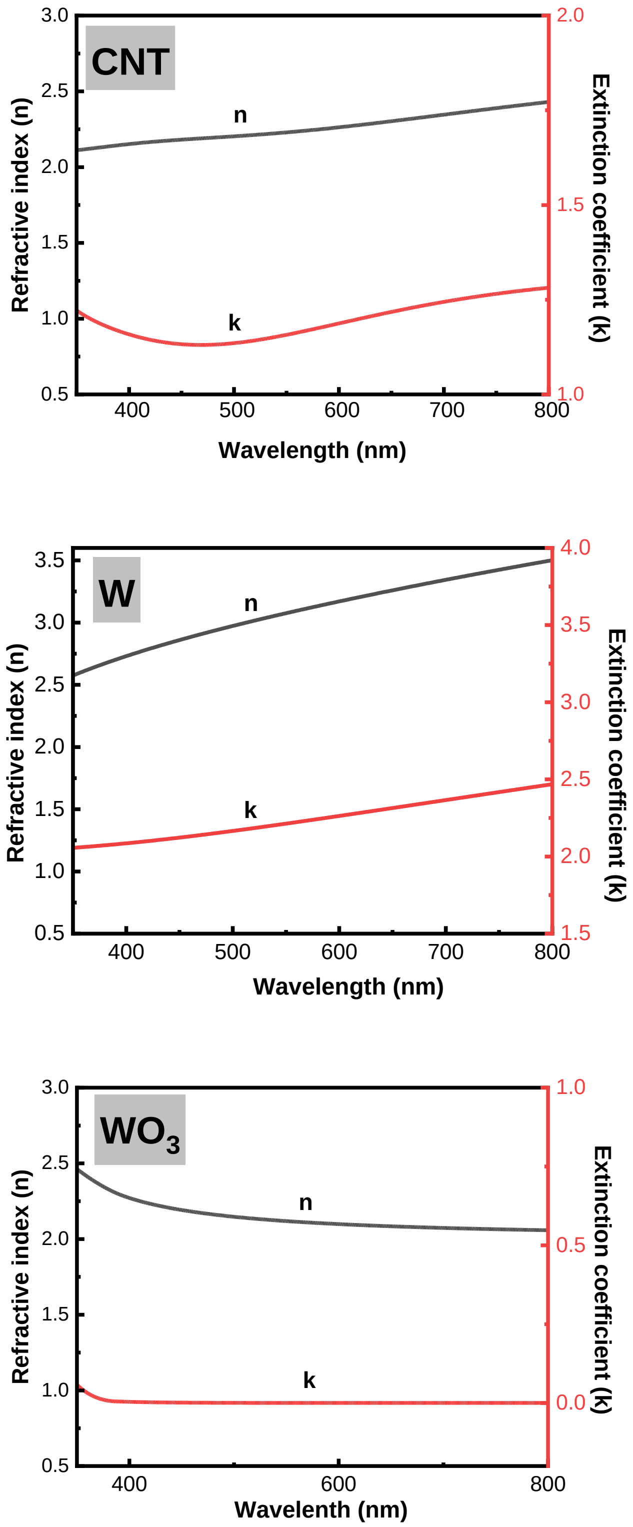

Figure S4. Refractive index (n) and extinction coefficient (k) of CNT, metallic W, and WO_3.

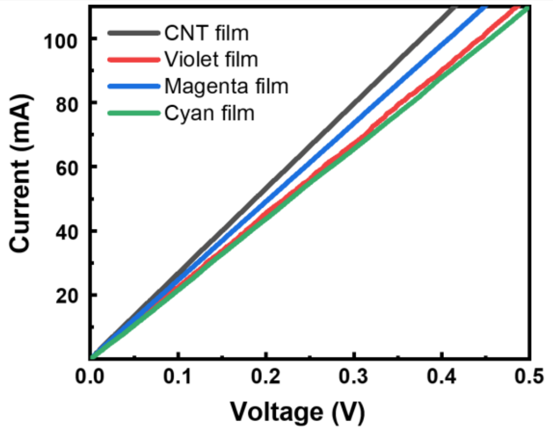

Figure S5. Current-voltage curves of CNT and CNT/W/WO_3 films.

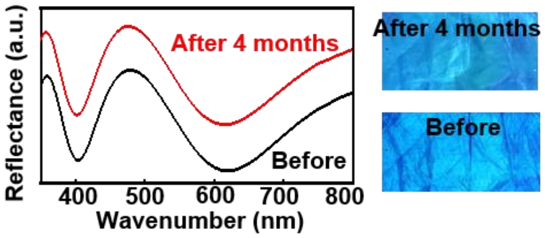

Figure S6. Tolerance of CNT/W/WO_3 to UV irradiation. Optical images and reflection spectra of CNT/W/WO_3 before and after UV irradiation.

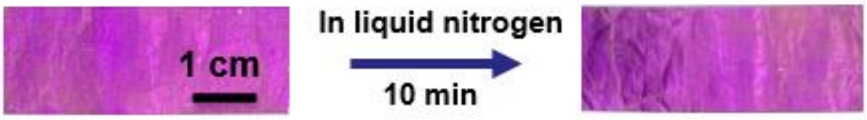

Figure S7. Optical images of CNT/W/WO_3 before and after exposure to liquid nitrogen.

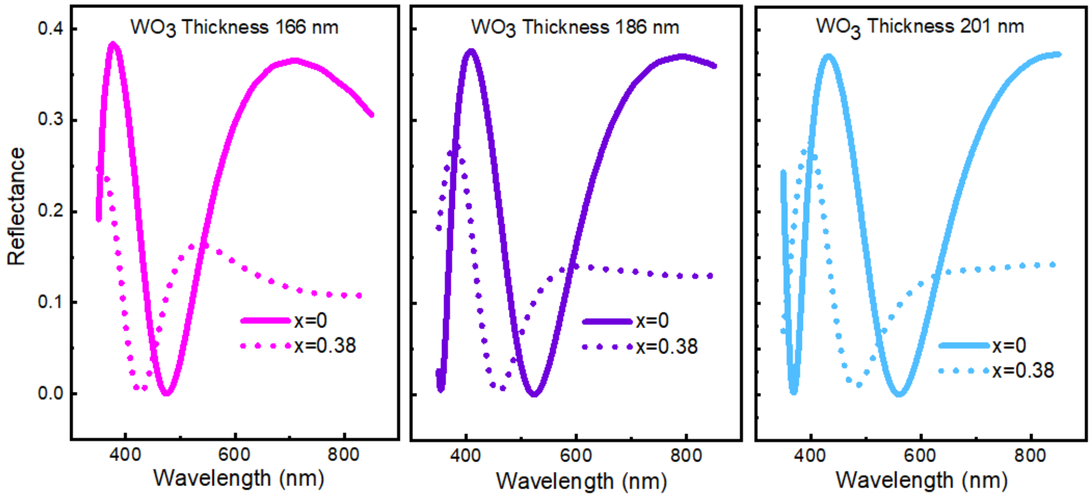

Figure S8. Reflectance spectra for x values of 0 and 0.38 for various thicknesses of tungsten oxide calculated by FDTD simulation.

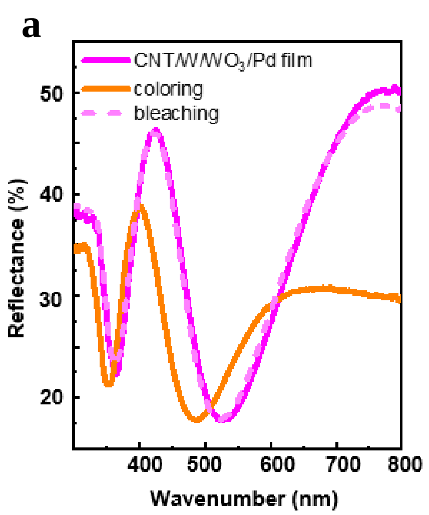

Figure S9. Reflectance spectra of (a) magenta and (b) violet film coloring and bleaching in pure H_2.

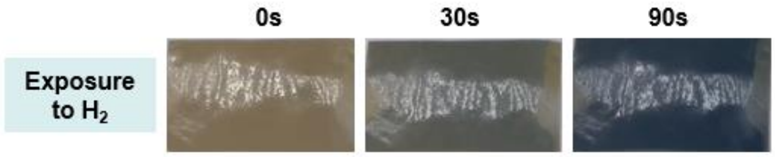

Figure S10. Optical images of color change with time for traditional WO_3/Pd film exposed to pure H_2.

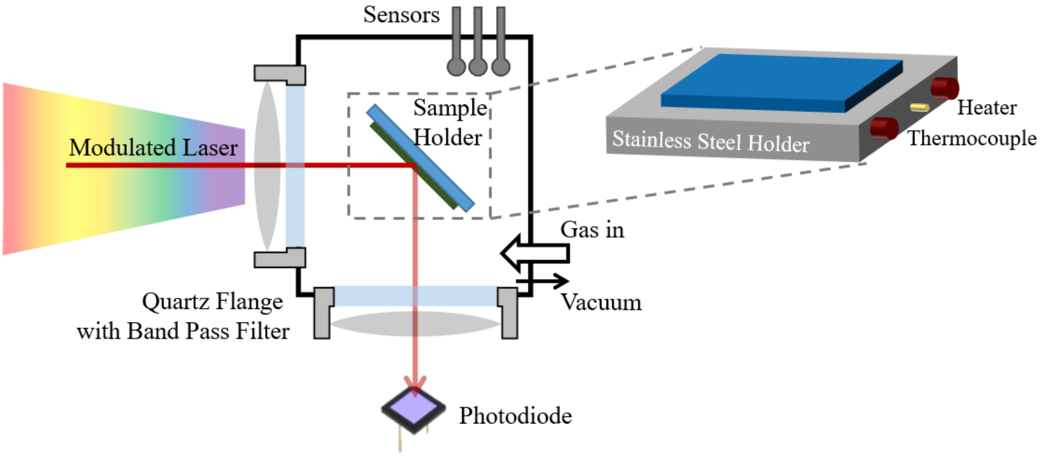

Figure S11. Schematic of test chamber and components used for optical testing. The actual equipment is shown in Figure 6 in [1].

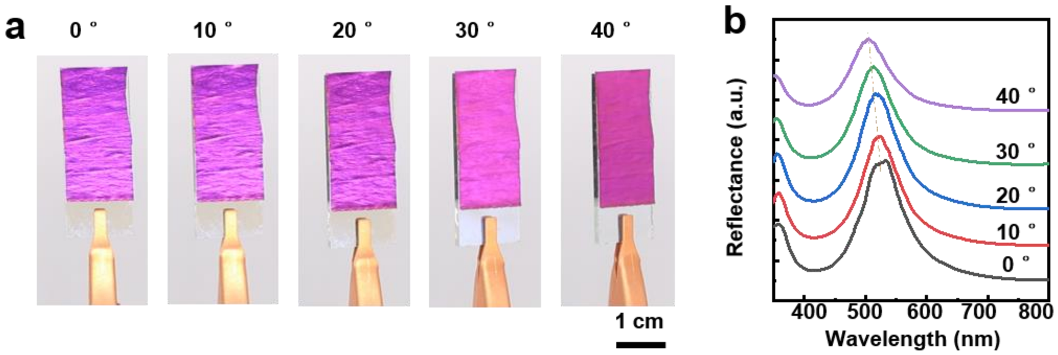

Figure S12. Angle sensitivity of W/WO_3-colored CNTs, a) Optical images from 0° to 40°, b) angle-resolved reflection spectra.

Supplementary Tables

Table S1. Color differences (ΔE_{ab}) between the three different colors of CNT/W/WO_3/Pd when exposed to pure H_2 for 30 s and 90 s relative to the original color and their corresponding L*a*b* values. (corresponding to Figure 3e).

CNT/W/WO$_3$/Pd Color	Coloring Time	L*	a*	b*	ΔE_{ab}
Blue	0 s	53.7	8	-58	-
	30 s	57	30	-52	**23.2**
	90 s	58	42	-38	**39.8**
Magenta	0 s	68	49	-21	-
	30 s	67	37	8	**31.4**
	90 s	70	19	31	**60.1**
Violet	0 s	54	42	-71	-
	30 s	54	60	-69	**18.1**
	90 s	52	66	-56	**28.4**

Table S2. Reflectance change, resulting color difference and corresponding L*a*b* values for blue CNT/W/WO_3/Pd (corresponding to Figure 3g).

CNT/W/WO$_3$/Pd Color	Coloring Time	L*	a*	b*	ΔR (%)	ΔE_{ab}
Blue	0 s	53	8	-58	-	-

	6 s	52	18	-57	20	**10.1**
	23 s	49	30	-56	38	**22.4**
	44 s	49	31	-50	42	**24.7**

Table S3. Corresponding to Figure 3h gasochromic performance curves, the values of reflectance change (ΔR) and coloring and bleaching times at different concentrations of hydrogen.

**H_2 concentrations in H_2 / Air**	**0.1%**	**0.3%**	**0.6%**	**0.9%**	**1.2%**	**1.5%**	**2%**
**ΔR (%)**	7.24	15.92	28.31	39.39	52.57	61.25	66.38
**$\Delta R_{90\%}$ (%)**	6.52	14.33	25.48	35.45	47.31	55.13	59.74
**Coloring Time （$\Delta R_{90\%}$） / s**	366	426	438	398	468	445	448
**Bleaching Time （$\Delta R_{90\%}$） / s**	132	138	156	175	256	292	344
**Coloring Time （Naked eye ΔR） / s**	-	-	58	20	9	7	6

Table S4. Comparison of the color difference between the film color at the leak point and the surrounding film color at 30 s and 180 s of hydrogen leakage and the corresponding L*a*b* values (corresponding to Figure 4c, e, f).

**CNT/W/WO_3/Pd Color**	**Coloring Time**	**L***	**a***	**b***	**ΔE_{ab}**
**Blue**	0 s	48	23	-82	-
	30 s	46	52	-84	**29.1**
	180 s	47	66	-83	**43**
**Magenta**	0 s	53	55	-3	-
	30 s	59	45	10	**17.5**
	180 s	64	25	28	**44.5**
**PET**	0 s	58	22	-45	-
	30 s	58	25	-44	**3.2**

180 s	56	29	-38	**10.1**

Table S5. Sputtering parameters of fabricated CNT/W/WO_3 films.

CNT/W/WO_3 Color	WO_3 Thickness (nm)	Gas Flow Ar/O_2 (sccm)	Vapor depositing Time (s)	Power (W)
**Magenta**	166	81/27	475	100
**Orange**	154	81/27	445	100
**Yellow**	135	81/27	390	100
**Cyan**	213	81/27	615	100
**Green**	219	81/27	630	100
**Blue**	210	81/27	607	100
**Violet**	186	81/27	535	100

Table S6. Vapor deposition parameters for CNT/TiO_2 films.

CNT/TiO_2 Color	Baking Temperature (℃)	Gas Flow O_2 (sccm)	Vapor depositing Time (s)	Rate of Evaporation (Å/s)
**Violet**	200	80	1000	2
**Green**	200	80	1290	2
**Yellow**	200	80	1500	2

Supplementary Notes

(1) Reasons for choice of W layer on CNTs: as CNTs is known to be a highly absorbent material, in order to improve the quality of coloration, we introduce a reflective layer with high reflectivity. Considering that W has good cohesion interface with C [2] and high reflectivity, we first sputtered a very thin layer of W on the CNTs to increase the reflection. In the

manuscript Figure 2e we also compare the difference between W/WO_3 coloring with a W-reflecting layer and TiO_2 coloring, and it is clear from the photographic colors that the W-reflecting layer coloring is superior in brightness and chroma, which is confirmed by further spectral data. Therefore, we choose W as the reflective layer.

(2) Reasons for choice of W/WO_3 system: on the one hand, the W/WO_3 system can be prepared by magnetron sputtering, which has obvious advantages in scalable preparation compared with ALD coloring methods in previous studies [3]. On the other hand, in our previous work, it has been confirmed that W/WO_3 system can modulate structural colors with rich and wide gamut [4]. Therefore, the W/WO_3 system is chosen.

(3) Reasons for choice of WO_3: WO_3 has a high refractive index, which is advantageous for color modulation. WO_3 is a typical gasochromic material (for example, it can detect hydrogen), thus facilitating the dynamic color modulation of colorful CNTs. Therefore, WO_3 is chosen as the color modulation layer.

References

[1] J. Wei, M. Zhao, C. Wang, J. Wang, J. Ye, Y. Wei, Z. Li, R. Zhao, G. Liu, Y. Geng, R. Wang, H. Xiao, Y. Li, C. Li, Z. Gao, J. Gao, *Sensors* 2022, 22, 1014.

[2] Q.Q. Ren, D.Y. Dang, H.R. Gong, J.L. Fan, Y.M. Zhao, Carbon 2015, 83, 100-105.

[3] F. X. Chen, Y. Huang, R. Li, S. L. Zhang, Q. Y. Jiang, Y. X. Luo, B. S. Wang, W. S. Zhang, X. K. Wu, F. Wang, P. Lyu, S. M. Zhao, W. L. Xu, F. Wei, R. F. Zhang, Sci. Adv. 2022, 8, eabn5882.

[4] Z. Wang, X. Y. Wang, S. Cong, J. Chen, H. Z. Sun, Z. G. Chen, G. Song, F. X. Geng, Q. Chen, Z. G. Zhao, Nature Communications 2020, 11, 302.
